# Apert Syndrome - caveats of squint management

**DOI:** 10.22336/rjo.2023.35

**Published:** 2023

**Authors:** Rolli Khurana, Ankita Singh, Divya Kochhar, Shyam Sundar

**Affiliations:** * Department of Ophthalmology, Military Hospital (Ahmedabad), Gujarat, India; ** Department of Ophthalmology, Military Hospital (Bathinda), Punjab, India; *** Department of Ophthalmology, Armed Forces Medical College, Pune, Maharashtra, India; **** Department of Ophthalmology, Military Hospital (Roorkee), Uttarakhand, India

**Keywords:** Apert Syndrome, craniosynostosis, inferior oblique overaction, V pattern strabismus

## Abstract

Apert Syndrome (AS) is a rare form of acrocephalosyndactyly. The aim of the manuscript was to underline the challenging squint management in a case of Apert Syndrome. A 1.5-year-old male with craniosynostosis, diagnosed at birth, with history of incomplete closure of eyes, more so in the right eye, and squinting of left eye since birth, was brought to eye OPD by the mother. Presence of acrocephaly, prominent forehead with bony irregularity, chin down with left head tilt, fused cervical vertebrae, marked proptosis, cleft palate, dental anomaly and syndactyly confirmed the diagnosis of AS. Old serial photographs of the child were examined to look for progression of squint and proptosis. Squint evaluation revealed 70-75 PD exotropia with 10PD right hypertropia in primary gaze. The right hypertropia increased further in the left gaze, whereas a left hypertropia was noted in the right gaze. The patient underwent bilateral LR recession of 9 mm with full muscle width transposition (upshift) with Inferior Oblique recession of 4:1 mm in the right eye and 3:2 mm in the left eye. Post-operative follow-up after 2 months showed that V pattern collapsed with residual exotropia of 20 PD. Post-operative follow-up after 1 year showed improvement in head posture with pattern collapsed. However, recurrent exotropia was noted on evaluation, for which bilateral medial recti resection was done later. The management of squint in AS and other craniosynostosis poses a multitude of challenges for the ophthalmologists. Frequent follow-ups are needed in patients with AS for the timely management of its ocular manifestations and better visual rehabilitation.

## Introduction

Apert Syndrome (AS), a rare form of acrocephalosyndactyly, which was first described by Wheaton in 1894, is a congenital abnormality characterized by craniosynostosis, midface hypoplasia, and syndactyly of limbs. The syndrome is named after the French pediatrician Eugene Apert, who first described nine people with similar characteristics in 1906 [**1**,**2**]. It is an autosomal dominant inheritance disorder with complete penetrance, but variable expression. The prevalence reported is 1 in 65000 live births with a total of 300 cases until 2019 [**3**]. The disorder occurs due to a gain of mutation in fibroblast growth factor receptor protein on chromosome 10q. Restricted anterior skeletal growth occurs due to early fusion of coronal sutures leading to shallow orbits and proptosis. Ocular features of AS included down-slanting palpebral fissure, hypertelorism, strabismus, proptosis, ptosis, lid malpositions, nasolacrimal dysfunction, refractive errors, papilledema, optic atrophy, and exposure keratopathy [**4**-**6**]. Strabismus is the most common association in patients with craniofacial dysostosis. V-pattern horizontal strabismus is the most common strabismus reported [**7**]. Excyclorotated orbits cause aberrant insertion or malrotation of extra ocular muscles leading to incomitant horizontal strabismus, most commonly exotropia [**7**,**8**]. Herein, we aimed to present the challenging squint management in a case of Apert Syndrome.

## Case presentation

**Fig. 1:**
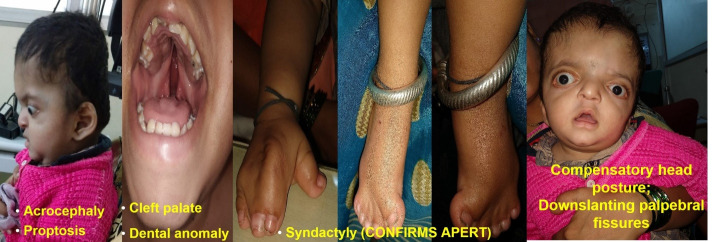
Picture showing the clinical features of Apert Syndrome present in the patient

A 1.5-year-old male infant, known case of craniosynostosis, diagnosed at birth, with history of incomplete closure of eyes, more so in the right eye and outward deviation of eyes since birth, associated with off and on watering and discharge, recurrent eversion of right lower lid and whitish discolouration in central clear part of both eyes in the last 2 months, was brought to the Eye OPD by his mother. Detailed antenatal history revealed that at the time of conception maternal age was 31 years and paternal age was 35 years. The mother offered a history of gestational Diabetes Mellitus, ischemic heart disease and polyhydramnios. Fetal Computerised Tomography scan (CT Scan) was done, but features of craniosynostosis were missed. The baby was born at full term through caesarean section. The infant underwent craniotomy surgery for acrocephaly at 9 months of age. They reported improvement in proptosis after cranial surgery. The parents also offered a history of recurrent microbial keratitis (treated) since he was 15 days of age. The presence of acrocephaly, prominent forehead with bony irregularity, chin down with left head tilt, fused cervical vertebrae, marked proptosis, cleft palate, dental anomaly and syndactyly confirmed the diagnosis of Apert Syndrome (**Fig. 1**). 

**Fig. 2:**
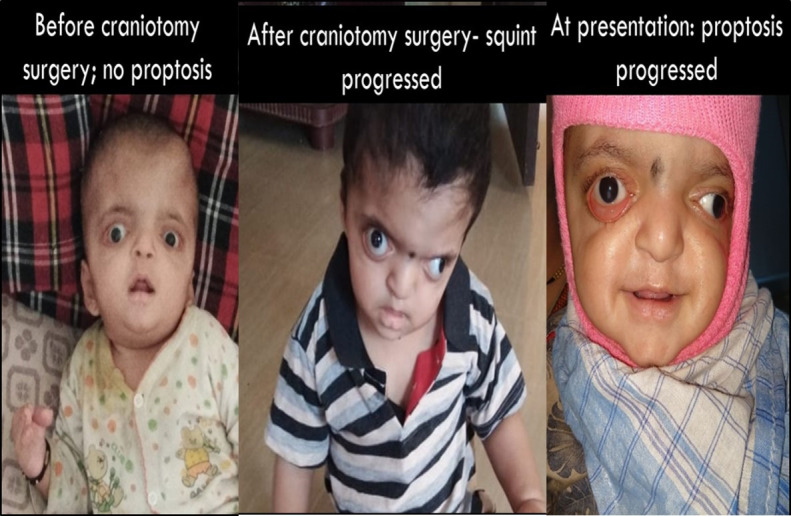
Serial photographs representing progression of proptosis and squint with craniotomy surgery and age

Ocular examination revealed fixing and following light uniocularly and binocularly with central, steady and maintained fixation, but with a poor attention span. Marked proptosis of 28 mm (R) and 26 mm (L), with a base of 98 mm, as measured by a Hertle’s exophthalmometer, was observed. A left head tilt with a compensatory chin up head posture was noted. Refraction of -0.5 DC X 170 in both eyes was recorded with retinoscopy done under cycloplegia. Torch light examination revealed downward slanting palpebral fissures, ectropion with lower lid laxity on both sides, fornix prolapse with chemosis and exposure keratopathy in both eyes. Orthoptic evaluation showed left exotropia with right eye preference. Ocular movements showed +1 elevation in adduction in both eyes. Excyclotorsion and disc pallor was ruled out by indirect fundoscopy. The patient was advised to wear right eye patching along with the administration of topical antibiotics and lubricants and vertical taping at night. Patching was continued for a duration of 1 year. Troublesome chemosis and fornix prolapse on follow up visits were initially managed conservatively. Later, the patient underwent lateral tarsorrhaphy on both sides for the same. Old serial photographs of the child were examined to look for progression of squint and proptosis with age related orbital changes and with craniotomy (**Fig. 2**). After craniotomy surgery, squint progression was noted. Since the moment of the initial presentation to the eye clinic, proptosis had also progressed.

**Fig. 3:**
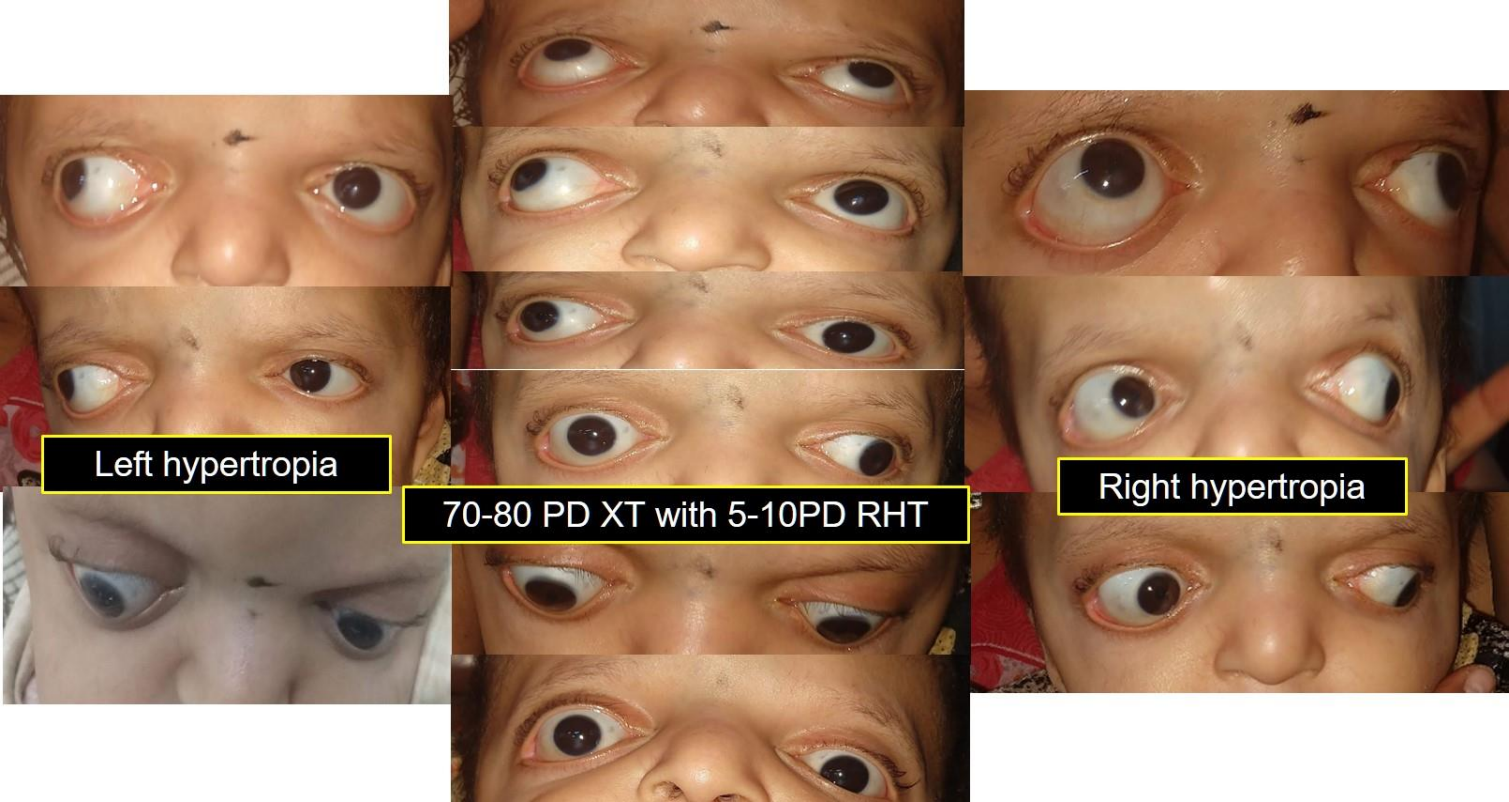
Squint evaluation in all the cardinal gazes revealing exotropia with V pattern

During the follow-up period, squint evaluation revealed 70-75 PD exotropia with 10 PD right hypertropia in primary gaze. The right hypertropia increased further in the left gaze, whereas a left hypertropia was noted in the right gaze. Also, the child was noted to be orthotropic in downgaze and developed a larger exotropia of around 100 PD in upgaze (**Fig. 3**). 

**Fig. 4:**
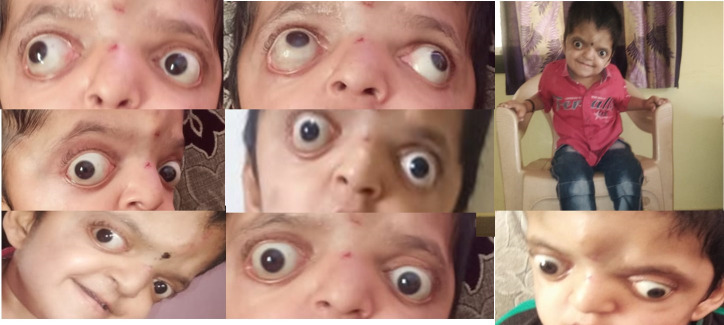
Post-operative photographs at 1.5 years, showing correction of V pattern and correction of chin up head posture

Exact measurements in gazes could not be taken as the child was uncooperative and hyperactive. MRI Brain and orbit was done, which ruled out the absence or abnormal course of superior oblique/ any extra ocular muscle. The patient underwent bilateral LR recession of 9 mm with full muscle width transposition (upshift), Inferior Oblique recession of 4:1 in the right eye and 3:2 mm in the left eye. The age of the child at the time of surgery was 2.5 years. Post-operative follow-up after 2 months showed that V pattern collapsed with residual exotropia of 20 PD. The patient was advised to continue alternate patching. Post-operative follow-up after 1 year showed improvement in head posture with pattern collapsed (**Fig. 4**). However, recurrent exotropia was noted on evaluation, for which bilateral medial recti resection was done later.

## Discussion

Craniosynostosis in Apert Syndrome may be detected in utero due to the presence of an aberrant skull shape [**9**] and may be further confirmed by family history and genetic testing for fibroblast growth factor gene mutation. In our case, despite undergoing a fetal CT scan, the findings of craniosynostosis were missed. Presence of a higher paternal age is also a known risk factor [**10**] for AS and was a positive finding in this case. 

Strabismus is a known ocular manifestation of AS. Excyclorotated orbits cause aberrant insertion or malrotation of extra ocular muscles leading to incomitant horizontal strabismus, most commonly exotropia [**7**]. There are multiple theories for the presence of V pattern in cases of AS. The mechanical hypothesis blames the antero-posterior shallowness of the orbit, which interferes with the normal acute 54-degree reflection of superior oblique tendon from the trochlea. This may reduce the efficiency of superior oblique action, allowing ipsilateral inferior oblique over action (IOOA) leading to V pattern strabismus [**7**]. Another theory suggests that a pseudo IOOA is created since the medial recti pull the eye up, whereas the lateral recti pull the eye down by the virtue of their shifted insertions in a malrotated orbit [**11**]. Moreover, the lack of inferior support to the inferior oblique in a shallow orbit may also cause an IOOA. In our case, we did not find any torsion on fundus imaging, thus ruling out the presence of true IOOA. Thus, we assumed shifted insertions of the horizontal recti to be the cause of V pattern in our case and upshifted both the lateral recti. However, in addition we also performed a bilateral inferior oblique recession since the V pattern was too large to be corrected by lateral recti transposition alone.

The management of squint in AS and other craniosynostosis poses a multitude of challenges for the ophthalmologists. In our case, surgical management was a challenge due to the left head tilt at all times as a result of cervical vertebrae fusion, down slant palpebral fissure - as a result of which appearance did not match with measurement, attention deficit aggressive child- making squint measurement even difficult, frequent associated ocular comorbidities and multiple systemic surgeries, which had already demotivated the parents, who did not understand the importance of strabismus correction. What was added to the agony was the changing proptosis and strabismus with cranial surgery and orbital growth. In fact, children may not present with proptosis at birth; the onset of symptoms may occur postnatally, which may progress over the first few years of life [**11**]. 

This makes the appropriate timing for surgical intervention for strabismus a controversial issue. The decision to delay surgery to wait for progression may be at the cost of loss of binocularity in such children. Various studies have advocated different timings of surgery in cases of AS. Morex et al. stated that 6 months to 1 year after cranial and mid facial surgery represents the ideal time for squint correction [**9**]. A study undergone by Diamond et al. showed that only 9 out of 140 cases had changes in squint after cranial surgery and summarized that early surgery should be done to restore binocularity [**12**]. Clement et al. stated that proptosis increases with age, changing the squint pattern. Hence, it is advisable to wait until later [**13**]. Miller et al. stated that 18-24 months of age is the ideal age for strabismus surgery [**14**]. Clement et al. also proposed that not only the surgery of the rectus muscle, but also the weakening of the oblique muscle should be performed to ensure an effective result [**13**]. In our case, the surgery was performed at 2.5 years old, that is, one year and 9 months after the craniotomy surgery. This age was selected in our case since the squint had been stable for the past 6 months and adequate occlusion therapy had been done to ensure free alternation of fixation between the two eyes.

## Conclusion

There are numerous theories pertaining to the etiology of V-pattern strabismus and oblique muscle dysfunction in patients with craniofacial dysostosis. The condition seems to be a result of complex anatomical anomalies. The associated strabismus complication might change patterns with the advancing age of patient, due to progressive bone alterations. Hence, sequential and titrated surgeries at least a few months after an early craniotomy surgery are advised for better ocular alignment and adequate visual and binocularity development. To conclude, frequent follow-ups, including age based visual acuity assessment and prescription of glasses along with management of amblyopia, orthoptic evaluation and management, appropriate prevention or treatment of exposure keratopathy and regular fundus examinations to monitor optic atrophy, are needed in patients with Apert Syndrome for a timely management of its ocular manifestations and better visual rehabilitation.

**Conflict of Interest**

The authors state no conflict of interest.

**Informed Consent and Human and Animal Rights statements**

Written informed consent has been obtained from the guardians to share the information, medical records, including pictures of the patient. 

**Authorization for the use of human subjects**

Ethical approval: The research related to human use complies with all the relevant national regulations, institutional policies, is in accordance with the tenets of the Helsinki Declaration, and has been approved by the institutional review board/ committee of Armed Forces Medical College, Pune, Maharashtra, India.

**Acknowledgements**

None.

**Sources of Funding**

None.

**Disclosures**

None.
